# The microRNA analysis portal is a next-generation tool for exploring and analyzing miRNA-focused data in the literature

**DOI:** 10.1038/s41598-021-88617-6

**Published:** 2021-04-26

**Authors:** Stefano Pirrò, Ivana Matic, Vittorio Colizzi, Andrea Galgani

**Affiliations:** 1 MirNat s.r.l., 00133 Rome, Italy; 2 Department of Biology, University of Rome Tor Vergata, Rome, Italy; 3 CIMETA, University of Rome Tor Vergata, Rome, Italy

**Keywords:** Data integration, Data mining, Data processing, Databases, Gene ontology, Gene regulatory networks, Literature mining, Software, miRNAs

## Abstract

MicroRNAs constitute a class of noncoding small RNAs involved in the posttranscriptional regulation of many biological pathways. In recent years, microRNAs have also been associated with regulation across kingdoms, demonstrating that exogenous miRNAs can function in mammals in a fashion similar to mammalian miRNAs. The growing interest in microRNAs and the increasing amount of literature and molecular and biomedical data available make it difficult to identify records of interest and keep up to date with novel findings. For these reasons, we developed the microRNA Analysis Portal (MAP). MAP selects relevant miRNA-focused articles from PubMed, links biomedical and molecular data and applies bioinformatics modules. At the time of this writing, MAP represents the richest, most complete and integrated database focused on microRNAs. MAP also integrates an updated version of MirCompare (2.0), a computational platform used for selecting plant microRNAs on the basis of their ability to regulate mammalian genes. Both MAP and MirCompare functionalities were used to predict that microRNAs from *Moringa oleifera* have putative roles across kingdoms by regulating human genes coding for proteins of the immune system*.* Starting from a selection of 94 human microRNAs, MirCompare selected 6 *Moringa oleifera* functional homologs. The subsequent prediction of human targets and areas of functional enrichment highlighted the central involvement of these genes in regulating immune system processes, particularly the host-virus interaction processes in hepatitis B, cytomegalovirus, papillomavirus and coronavirus. This case of use showed how MAP can help to perform complex queries without any computational background. MAP is available at http://stablab.uniroma2.it/MAP.

## Introduction

MicroRNAs (miRNAs) are noncoding, single-stranded small RNAs 18–24 nucleotides in length. They regulate gene expression through complete or incomplete complementarity with the 3ʹ-untranslated region (3ʹ-UTR) of target mRNA. It has been estimated that each miRNA targets multiple mRNAs, thus regulating almost 60% of human protein-coding genes^1^. The miRBase database^2^ (*miRBase 22.1 Release, October 2018*) contains 38,589 mature miRNAs in 271 species, including 2654 mature human miRNAs. Extensive microRNA-focused mining of PubMed articles showed that 68,087 were related to metabolism, 35,186 were related to cell development, 18,052 were related to apoptosis and 10,828 were related to cell differentiation. It is therefore no surprise that miRNAs play significant roles in the regulation of the pathological mechanisms of numerous diseases, such as cardiovascular diseases, obesity, diabetes and different types of cancer.

Research on miRNAs is one of the most widely discussed topics in science and medicine in the last decade. Bioinformatics tools and high-throughput sequencing contributed to the identification of numerous miRNAs and their potential gene targets. Therefore, the demand for monitoring scientific advancement and progress related to miRNAs is continuously increasing. It is estimated that the number of publications on miRNAs available on the PubMed platform will exceed 115,054 in 2021, with a continuous exponential increase trend evidenced thus far: in the first 10 years of miRNA discovery (in 2001), more than 10,000 articles have been published, and in the second decade, this number was ninefold greater. The rapid increase in the literature on miRNAs provides researchers with abundant information, making it difficult to accurately identify all available articles of specific interest and to keep up to date with the novel findings associated with miRNAs without getting overwhelmed with the flow of information.

The most efficient way to find articles on a topic is to search a database, allowing for browsing from hundreds of journals at one time. For example, Scopus^3^ is one of the two largest commercial bibliographic databases that cover scholarly literature from almost any discipline, together with Web of Science^4^. For the literature in medicine or biological sciences, PubMed is the number one resource: it stores abstracts and bibliographic details of more than 30 million papers and provides full text links to the publisher sites or links to the free PDF on PubMed Central (PMC). Despite the presence on the websites of other research databases (ERIC, ScienceDirect, Google Scholar, PubChase, and ReadCube), none combines the literature layer with molecular data or bioinformatic analyses.

A literature search is a key step in performing good authentic research and is helpful in formulating a research question and planning the study. On the other hand, an enormous quantity of available published data makes the perfect selection of appropriate articles relevant to a specific study almost an art. It is often time-consuming and tiring and can lead to disinterest or eventual renunciation of the search if it was not carried out in a stepwise manner. In this work, we present the microRNA Analysis Portal (MAP), which aims to provide a user-friendly platform to explore the most impactful literature linking microRNAs with selected topics of interest. MAP also includes an updated version of MirCompare (2.0), a computational platform for selecting plant microRNAs according to their ability to regulate mammalian genes^5^.

## Methods

**Figure 1:**
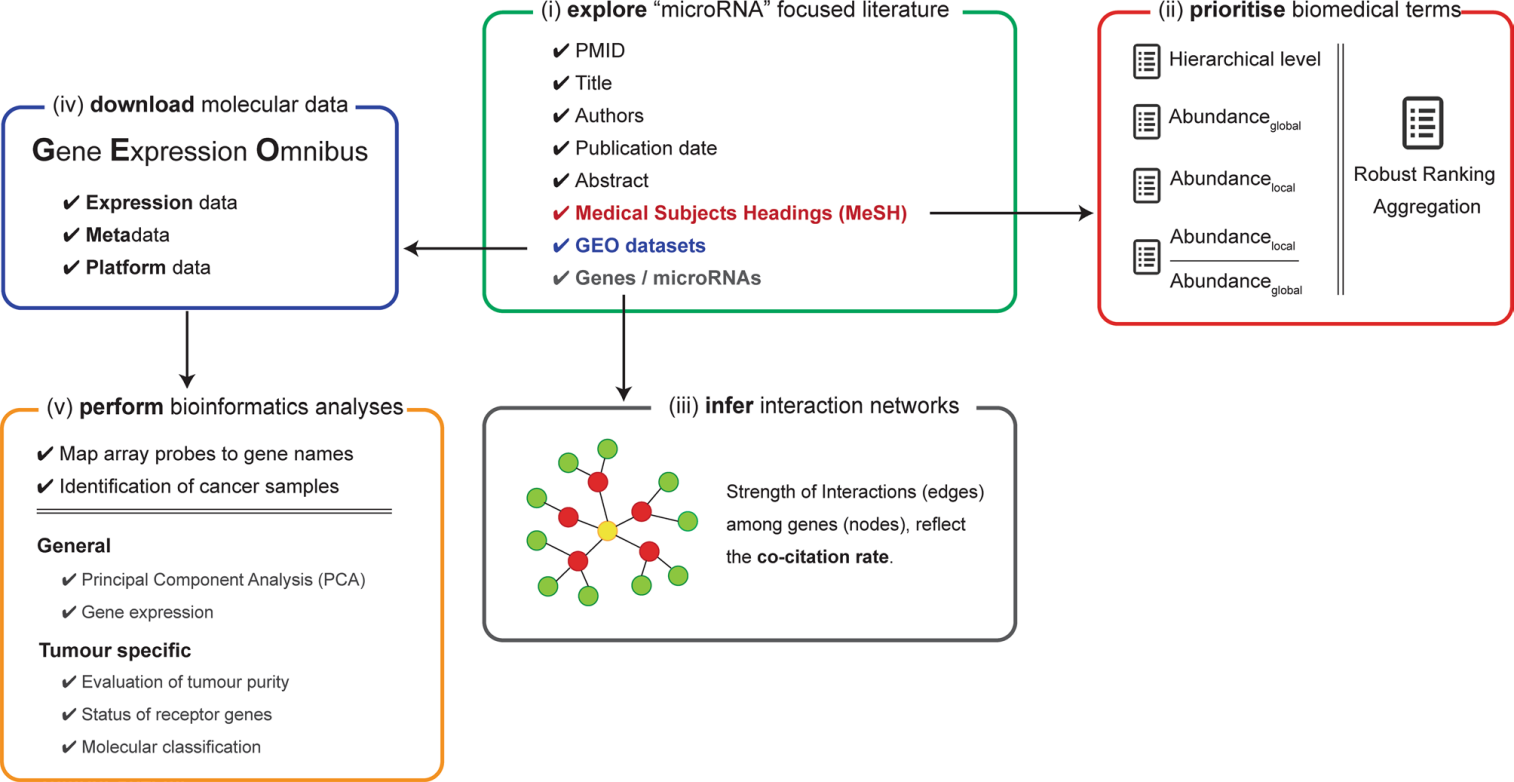
Overview of MAP update process.

MAP is powered by a custom (microRNA-focused) version of the Smart Automatic Classification system (SMAC)^6^. MAP has been designed for extrapolating, selecting and linking relevant articles from PubMed to biomedical and molecular data. MAP fully respects the architecture of SMAC for generating/updating datasets underlying the database and performs five main operations (Fig. 1): (i) exploring the literature (PubMed) by listing the most relevant manuscripts according to the “microRNA” query; (ii) extracting and prioritizing biomedical terms that enrich each manuscript; (iii) generating gene networks where strength and reliability of interactions is proportional to the cocitation rate; (iv) extracting array-based expression data from Gene Expression Omnibus (GEO)^7^ and converting it into a standard format; and (v) performing a range of bioinformatics analyses, selected according to the phenotype of the analyzed sample. To the best of our knowledge, MAP represents the most complete and extensive collection of data tuples (*PubMed articles–GEO data–Bioinformatics analyses*) associated with the microRNA field of study. The rapidity and flexibility of the MAP engine opens up the opportunity for periodic enrichment of our resource with minimal manual intervention.

### Generate and explore the literature collection

MAP collects and organizes manuscripts that link microRNAs to select organisms (20), diseases (11,376—extracted from Malacards^8^) and miRNA names (48,916—extracted from miRbase^2^). For each element of a linkage, a search query is constructed, and all the information is then collected (i.e., PMID, title, authors, abstract). Every downloaded paper is then enriched with a set of medical subject headings (meSH) and gene identifiers.

The “Explore” section of the MAP portal presents a snapshot of up-to-date manuscripts derived from the literature collection procedure. Users have an opportunity to filter articles according to a mixture of general features (keyword, publication date, author, journal), organisms, meSH records, genes and microRNAs of interest.

**Figure 2:**
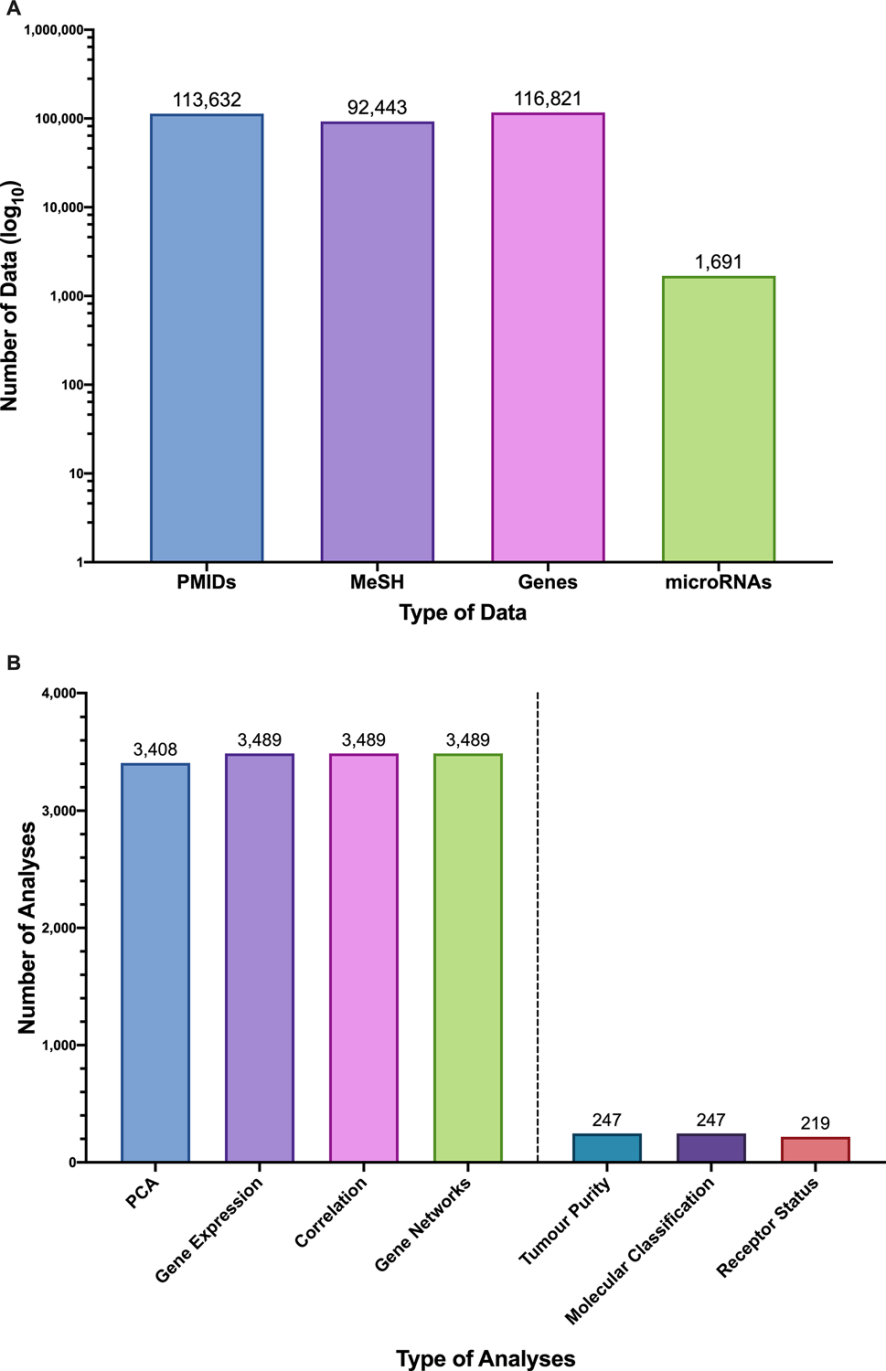
Statistical report of total number of data (**A**) and analyses (**B**) stored in MAP. Bioinformatics analyses have been separated in general (left) and cancer-specific (right).

As reported in Fig. 2A, the current version of MAP collects information from more than 113,000 manuscripts and finds connections among 116,821 genes, 1,691 microRNAs and 92,443 MeSH terms.

### Extract molecular data

As mentioned above, many of the records in MAP are connected with molecular datasets deposited by authors in the GEO database. In this regard, the main limiting factor is the absence of deposited data. MAP breaks the barrier of data inconsistency when attempting to access data programmatically; however, this advantage often leads to data quantity being sacrificed to ensure high quality data are obtained.

The integration of molecular data generated from published studies relies on the Python package GEOparse (https://GEOparse.readthedocs.org) to retrieve expression datasets from the NCBI Gene Expression Omnibus^7^. For each GEO series (GSE), three data packages are generated to reflect sample-level granularity.

*pData* include the phenotypic and experimental information deposited by a research group. A text-mining approach is applied for stratifying samples into different biological groups. Moreover, cancer samples are identified and separated from normal/control samples. This step is crucial for performing a subset of analyses particularly designed for tumor data.

*eData* packs the expression levels related to each sample.

*tData* contains a conversion dictionary of probes and gene names. This is a key requirement to reduce the dimensionality of the expression dataset because it merges expression levels related to the same gene, thereby facilitating subsequent bioinformatics analyses.

Figure 2B reports the total number of analyses that have been performed on MAP datasets, classified by type. The option of publication filtering on the basis of the presence/absence of associated GEO datasets is provided.

### Perform bioinformatics analyses

Once a paper with associated data is selected, the user has access to several exploratory and investigative bioinformatics analyses. If the selected dataset contains tumor-derived samples, additional cancer-specific analyses are launched automatically (Table 1).

**Table 1:** Description of bioinformatics analysis available in MAP.

Category	Name	Description	Dependencies	Display
Core	Principal Component Analysis (PCA)	Identification of key components of variability in the expression data	Plotly	Scatterplot (2D, 3D), barplot
	*Gene specific* Expression profile	Gene-centric expression profile summarized across samples and biological groups	ggplot2	Boxplot, barplot
	*Differential expression* profile	Expression profile for top 100 differentially expressed genes, across all samples and groups	ggplot2	Heatmap
	Gene correlation	Pearson correlation among subset of genes, across all samples and groups	ggplot2, heatmaply, plotly	Heatmap
	Gene network	Interactions between genes of interest and their primary neighbours. Based on the interactome dataset MENTHA^81^, overlaid with the expression data summarized across the groups	visNetwork	Interaction plot
	Functional enrichment	A statistical test for determining the enrichment of biological functions in the list of most perturbed genes	ClusterProfiler	DotPlot, Heatmap, UpSet plot
Cancer specific	Tumour purity	Estimate tumour purity and the presence of infiltrating stromal/immune cells for each sample	Estimate	Scatterplot (3D)
	Molecular classification	Classification of tumour samples according to PAM50 predictor model	Plotly, genefu	Barplot
	Receptor status	Stratification of cancer samples according to the expression level of ER, PR and HER2 receptors	Plotly, mclust	Barplot

Data are processed using a standardized workflow to ensure comparability, reusability and interoperability across different datasets and different data types. For instance, gene expression data extracted from GEO undergo z-score transformation to ensure that the data from different studies are presented at the same scale.

Most results are presented in an informative graphical format using the visualization library Plotly^9^. All the statistical and scientific charts can be interactively explored by visualizing the annotation of data points, zooming to focus on the area of interest, excluding/including subgroups in the data, and downloading the results as static image files of publication quality. Where applicable, the results are also presented in tabular format with filtering, pagination, and sorting options and are available for download in multiple formats.

### Principal component analysis

Principal component analysis (PCA)^10^ can be considered the initial step to conduct exploratory analyses on expression data. The aim of this technique is to reduce the dimensionality (complexity) of the dataset, increasing the interpretability while minimizing information loss. Data are transformed into a coordinate system that maximizes the variance among the features in the original dataset and then are presented in orthogonal projections. PCA is a powerful method for obtaining a global view of the data structure and identifying key ‘components’ of variation.

MAP shows 2D and 3D scatterplots representing the first two and three principal components (PCs), respectively. Additionally, to provide an overview of the global variability for the selected dataset, the fraction of total variance attributed to each PC is also provided. For exploratory analysis, PCA captures the presence of clusters of samples showing similar expression patterns. Indeed, the position of samples (dots) reflects their mutual similarity.

### Evaluation of gene expression

Two types of inquiries focused on the evaluation of gene expression can be conducted through the MAP user interface. The *gene-specific* feature tracks the normalized expression levels (z-scores) for a gene of interest across all the samples in the dataset. To provide a comprehensive overview of the distribution of values across several biological conditions, the results are presented in the forms of bar plots and boxplots. The bar plot summarizes the expression values by representing the mean and standard deviation (y-axis) for each biological group (x-axis). The box plot boosts data granularity by showing gene expression level quartiles (y-axis) in samples, stratified according to their group (x-axis).

In the *differentially expressed* section, the normalized expression levels (z-scores) for most variable genes (n = 100) are presented across all samples in the GEO dataset. Moreover, samples are clustered according to the resulting expression profile. This analysis produces a heat map where rows and columns represent genes and samples, respectively.

### Correlation among genes

The Pearson product moment correlation coefficient (PMCC) is applied to define relationships between user-defined genes (at least 2 and up to 50) in the same dataset.

The result of this analysis is shown as a heat map, wherein the color of each cell indicates the correlation coefficient between corresponding genes labeled on the x-axis and y-axis. The heat map color key is displayed on the right side of the plot, with red and blue indicating high and low correlation values, respectively. The calculated correlation coefficient and p-value can be visualized for each pairwise comparison by hovering over the heat map.

### Gene interaction and functional enrichment

For assessing the up/downregulation of selected genes, gene interaction networks and functional enrichment are two crucial explorative analyses that allow us contextualize perturbations to cell functionalities. For each biological group defined in the dataset, the interactions between genes of interest and their primary neighbors are displayed in an interactive network in which genes (nodes) are colored according to their normalized (z-score) expression.

This feature is powered by the Mentha interactome browser^11^, which collects manually curated interactions from databases that have adhered to the IMEx consortium curation guidelines^12^. A detailed report of the interactions composing a network, together with the list of PMIDs that support each relationship, is also provided to the final user in tabular format.

Whenever possible, MAP also applies overrepresentation analysis (ORA)^13^ using the Gene Ontology^14^, KEGG^15^ and REACTOME^16^ databases to determine whether known biological functions are enriched in the experimental setting. Each enrichment is summarized in both graphical (dot plot, heat map and upset plot) and tabular formats.

### Estimation of tumor purity

It is well known that malignant solid tumor tissues consist of an unbalanced mixture of tumor, stromal, immune and vascular cells. The combination of these cellular types has a strong influence on tumor growth, progression^17,18^ and drug resistance^19^. Evaluating the accurate tumor content in a cancer sample represents an open, scientific challenge and may provide important insights into the development of robust diagnostic and predictive models.

Starting from array-based expression data, MAP applies the ESTIMATE algorithm^20^ to infer tumor purity from previously calculated stromal and immune scores. To facilitate the comprehension of the scores and their correlation with the inferred tumor composition, all the values are aggregated and represented in a single, interactive scatterplot. Related values are also reported in a dynamic table for use in further filtering procedures.

### Status of receptor genes

Estrogens are steroid hormones that exert pivotal effects on the reproductive and gastrointestinal systems, mammary glands, skeletal and immune systems, and even the central nervous system^21,22^. Progesterone plays roles in the regulation of several reproductive processes, including ovulation and sexual behavior^23^. Human epidermal growth factor-2 receptor (HER2) is a member of the epidermal growth factor receptor family with tyrosine kinase activity and is involved in cell growth under normal conditions.

The evidence that estrogen and progesterone hormones are involved in cancer is overwhelming. In breast cancer, they interact with counterpart receptors (ER and PR) to promote cell proliferation by inducing cyclin G1 expression^24^ HER2 its overexpression leads to mammary adenocarcinoma in a single step, highlighting this receptor as the main driver of carcinogenesis in certain tissues^25^. HER2 overexpression has also been seen in other cancers, such as the ovary, endometrium, bladder, lung, colon, and head and neck^26^.

In recent years, the correlation between ER, PR, HER2 and clinical outcomes in other types of cancers has been explored^27^. In 2018, Wang and coauthors shed light on the prognostic value of progesterone receptors in solid pseudopapillary neoplasms of the pancreas and confirmed that a negative PR was significantly associated with poorer disease-free survival (DFS) and disease-specific survival (DSS)^28^.

A recent work from Chou and coworkers found that HER2-amplified pancreatic ductal adenocarcinomas (PDAC) have an atypical pattern of metastatic spread with a predilection for lung metastasis and local recurrence, but not liver metastases^29^.

In this context, MAP stratifies cancer samples according to the expression levels of ER, PR and HER2 receptors. Gaussian finite mixture modeling is applied to the expression data, and each sample in the dataset is categorized accordingly. Subsequently, samples that are negative for all the receptors (triple negative) are identified and highlighted. Each classification outcome is shown as a stacked bar chart.

This analysis aims to simplify investigations about the relationships between selected genes/microRNAs and a tumor subtype.

### Molecular classification

During the past decade, the development of gene expression signatures with prognostic and diagnostic value has become essential in precision medicine in oncology. In 2000, 4 distinct subtypes of breast cancer with clinical implications were identified from microarray gene expression data^30^. Almost a decade later, Parker and coworkers developed a 50-gene signature (PAM50) for subtype-based stratification^31^.

Only a few years later, the PAM50 assay was used to develop a model for predicting the tumor growth rate^32^ and is now a tool used daily for assessing the indication for adjuvant chemotherapy^32,33^.

In addition to its established role in breast cancer, the PAM50 classifier has been successfully applied to bladder^34^, lung^35^ and prostate^36^ cancer, wherein these classification systems assume a high-grade predictive value^37^.

MAP applies the PAM50 predictor model for the classification of tumor samples and reports the results in both graphical and tabular formats.

### Prediction of cross-kingdom miRNAs

A section on the web interface of MAP is dedicated to MirCompare (version 2.0), a bioinformatics tool developed by our group in 2016^5^. MirCompare uses libraries of miRNAs belonging to organisms from plant and animal kingdoms to find cross-kingdom functional homologies.

Analyses are submitted in the background to a dedicated server respecting a queueing system. When the analysis is completed, the results are sent to the final user by email to be downloaded, visualized and (eventually) further analyzed.

### Sequence alignment

The local alignment accounts for open and extended gaps in the global (whole sequence) and local (seed-specific) alignments.

In accordance with our previous version, the global alignment score between two sequences *S*_*A,B*_ is defined as the number of matches with respect to the maximum length (1). For this reason, the global alignment assigns + 1 to a case of matching and 0 otherwise.

1 $${S}_{A,B}= \frac{{matches}_{A,B}}{max\left({length}_{A}-{length}_{B}\right)}$$

The seed-specific alignment is needs to much more stringent than the global alignment, and penalties have been assigned to mismatches (− 0.5), open gaps (− 1) and extended gaps (− 1). According to previous studies^38–40^, this approach valorizes comparisons with a very strong homology in the seed region with respect to other sites.

### Assessing the statistical significance of each comparison

**Figure 3:**
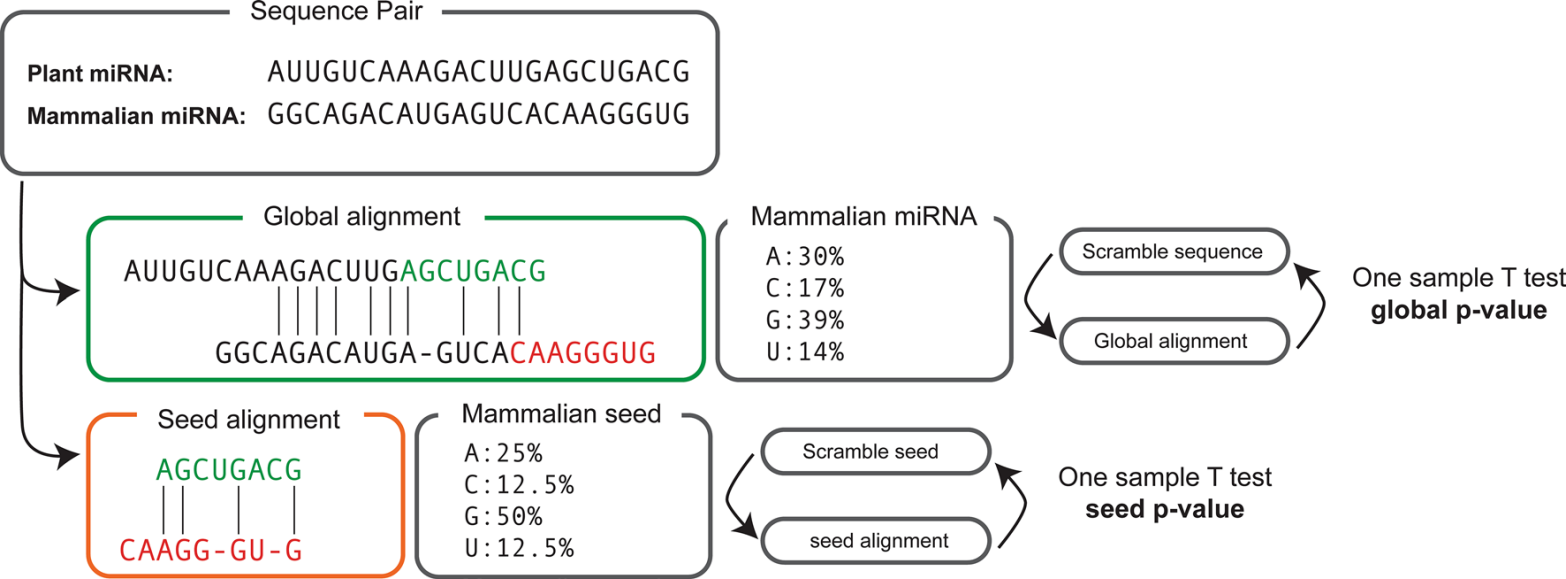
Inferring statistical significance for pairwise comparisons.

As reported in Fig. 3, given two sequences A (from plants) and B (from mammals), we assess whether the magnitude of the comparison is far from random.

We determine the percentage of nucleotides in the B sequence, and we generate 100 scramble sequences (B’) accordingly. Then, a series of 100 *S*_*A,B’*_ are calculated, and a one-sample T test is performed. This approach is applied to both the global (whole sequence) and local (seed region) alignments, resulting in two distinct p-values for each comparison.

### Selection of experimentally validated target genes and prediction of new genes

After a list of comparisons between plant and mammalian microRNAs is generated, MirCompare identifies the target genes in the host species. To perform this operation, it is assumed that plant microRNAs regulate host mRNA translation in a manner analogous to their mammalian counterparts.

The first selection step involves the selection of genes experimentally shown to interact with a pool of select microRNAs. For this purpose, MirCompare queries DIANA-TarBase v.8.0^41^, the most up-to-date collection of experimentally supported interactions between microRNAs and targets. The retrieved records are then ranked according to the robustness of the supporting methodologies and presented to the final user in the form of a TSV (tab-separated) file.

The second step consists of the in silico prediction of new putative target genes. To this aim, we use ComiR^42^, an algorithm based on a support vector machine (SVM), to combine the predictive power of four popular scoring systems (miRanda^43^, PITA^44^, TargetScan^45^ and mirSVR^46^). Since this process can be very long, we precomputed the targets for *H. sapiens* and *M. musculus.*

### Functional enrichment analysis

**Figure 4:**
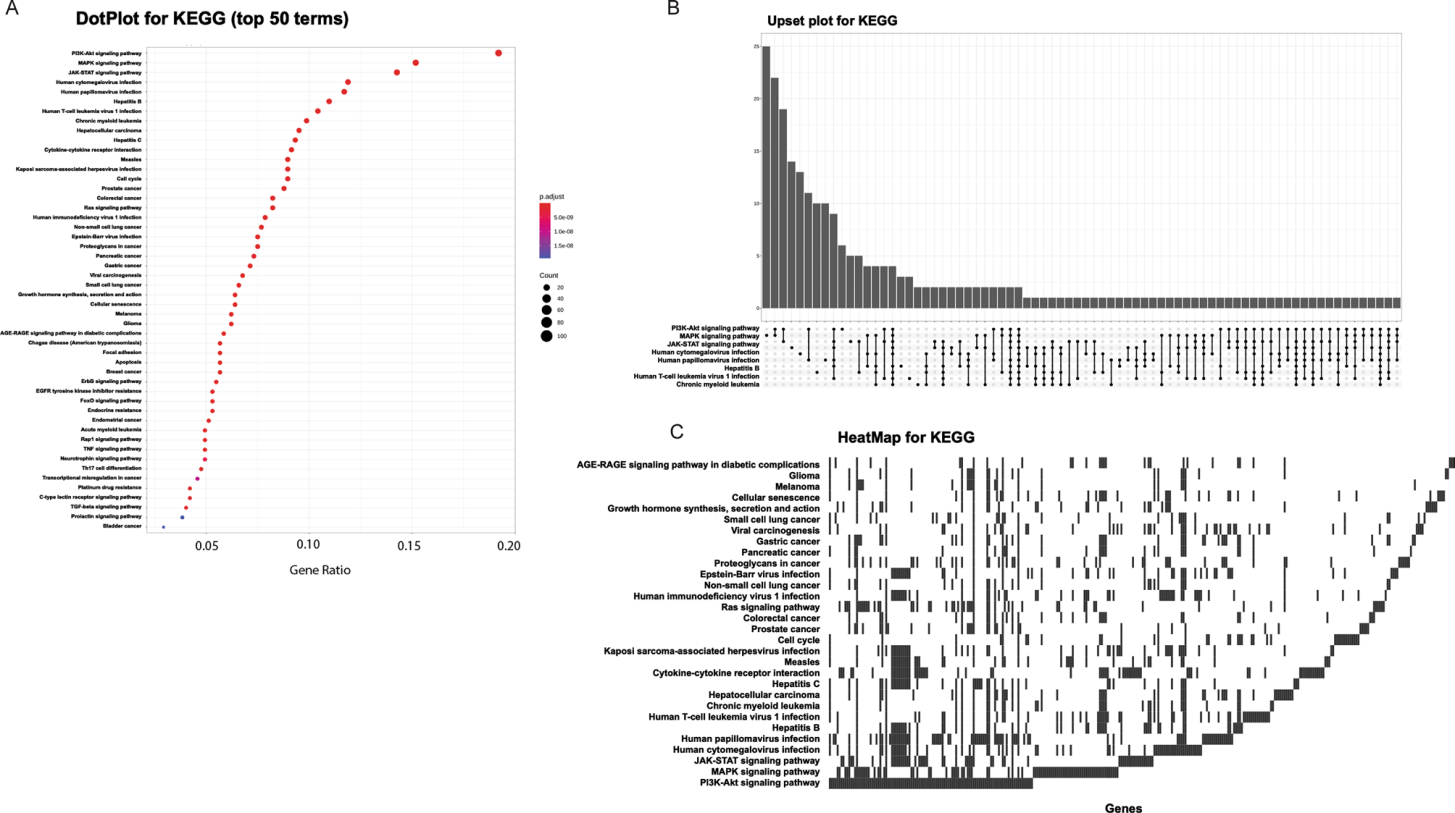
Functional enrichment analyses in MAP and MirCompare are visualized as dot plot (**A**), upset plot (**B**) and heatmap (**C**). Visualizations have been generated by using the clusterProfiler^48^ R^47^ package (sourcing KEGG^15^ database).

Starting from the list of genes that are putatively targeted by plant miRNAs, a central overrepresentation analysis (ORA)^13^ is applied to determine whether known biological functions or processes are overrepresented (enriched) with respect to the background. A p-value is also calculated by hypergeometric distribution and adjusted for multiple comparisons. MirCompare uses the R^47^ package ClusterProfiler^48^ for enquiring many different ontologies and signatures (WikiPathways^49^, MSigDB^50^, Disease Ontology^51^, Network of Cancer Genes^52^, DisGeNET^53^, Gene Ontology^14^, KEGG^54^, and REACTOME^16^). The results of each enrichment are summarized in both graphical and tabular formats. A dot plot reports the top 50 enriched terms, correlating GeneRatio, number of genes and p-value (Fig. 4A); the UpSet plot emphasizes the gene overlapping among different gene sets (Fig. 4B); the heat plot displays the relationships between genes and terms as a heat map, simplifying the identification of patterns (Fig. 4C).

## Results

### Identification of *M. oleifera* microRNAs involved in immune processes

According to MAP, more than 2000 research papers have been published on the role of microRNAs in immune system regulation and inflammatory processes. MiRNAs act on all levels of the immune system, from hematopoietic development to activation in response to infection, during both innate and acquired immunity.

The immune system ensures complex and well-orchestrated protection against pathogens to which an organism can be exposed. The initiation, propagation and resolution of every response must be carefully coordinated and balanced; otherwise, the positive result of an immune response can be hampered or can lead to chronic conditions/diseases. The immune response heavily relies upon a predetermined program of DNA rearrangements in lymphocytes as main actors, and microRNAs are important regulators of intricate systems. The first report on the involvement of miRNAs in immunity was the identification of multiple miRNAs specifically expressed in hematopoietic cells^55^. Moreover, hematopoietic cells can be selectively identified by their miRNA expression profile: they all express five highly specific miRNAs, miR-142, miR-144, miR-150, miR-155 and miR-223^56^, and distinct lineages of immune cells can also be distinguished by their unique miRNA expression profiles: erythrocytes show higher expression of miR-451, whereas B and T lymphocytes express miR-223^57,58^.

With respect to the regulation of an inflammatory response, the most studied actor is miR-155, which is induced early in macrophages as a consequence of exposure to a broad range of inflammatory mediators^59^.

Another important example is miR-16, which targets mRNAs for ARE-mediated degradation^60^, thus influencing the temporal order of the induction of genes encoding inflammatory molecules^61^. miR-16 is involved in the regulation of various types of infection: tuberculosis^62^, malaria^63^, and enterovirus infections^64^.

In our previous studies^65,66^, we focused on the characterization of microRNAs from *M. oleifera,* a medicinal plant widely distributed in subtropical areas and popularly called the “miracle tree”. Although the anti-inflammatory and immunomodulatory properties of this plant have been widely studied and associated with polyphenols^67^, the role of microRNAs in this context has been underexplored. In 2019, we identified a set of *high-confidence* (hc, 131), *low-confidence* (lc, 300) and *novel* (n, 302) microRNAs from the leaves, seeds and calli under normal and cold-stress conditions^66^.

Among several miRNAs, hc-mol-miR159 was highly expressed under all experimental conditions and is also one of the most conserved plant microRNAs. Interestingly, miR-159 has been proven to be actively involved in cross-kingdom regulation of the TCF7 gene in humans^68^. The levels of this microRNA in human serum are inversely correlated with breast cancer incidence and progression.

Starting from these insights, we took advantage of both MAP and MirCompare to identify microRNAs from *M. oleifera* putatively involved in cross-kingdom regulation of immune processes.

### Selection of immune-related microRNAs in humans

**Figure 5:**
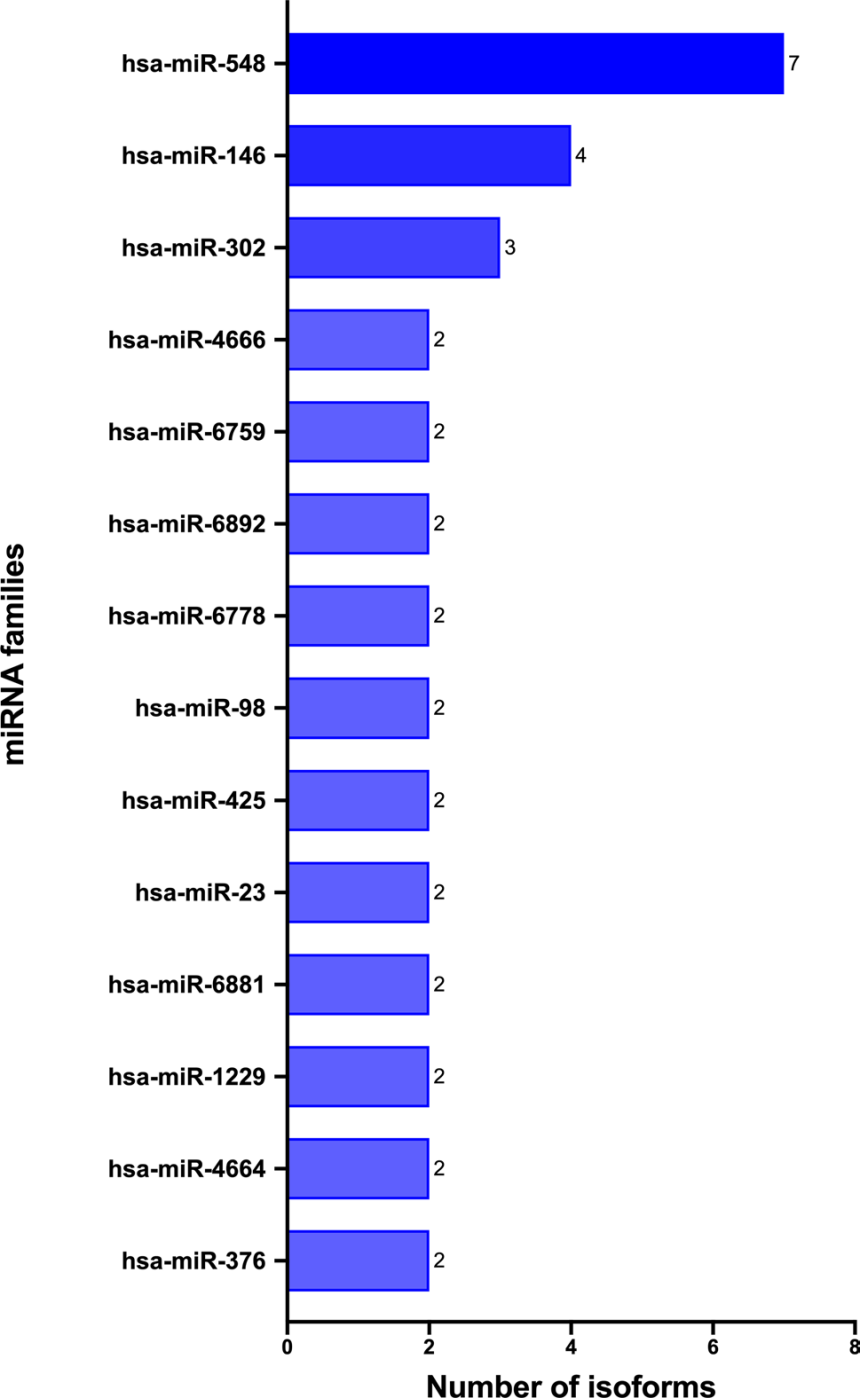
Abundance of isoforms for human microRNAs correlated with immunity topic in MAP.

The first step of our analysis consists of using the literature module of MAP to list the human microRNAs associated with immune-related MeSH terms. Performing this exploratory analysis in MAP is fast and easy and requires only a few clicks. Starting from a recovered total of 1001 publications and 1054 microRNAs in 22 different species, we filtered 94 human sequences (Supplementary Table 1). As reported in Fig. 5, the most abundant miRNA family is hsa-miR-548 (7 isoforms), followed by hsa-miR-146 (4 isoforms) and hsa-miR-302 (3 isoforms). Exploring the relationship between miR-548 isoforms and immunity in more detail, we discovered that its involvement in the regulation of the host antiviral response via direct targeting of interferon (IFN)-mediated pathways has been well elucidated^69–71^.

### Functional homology between *M. oleifera* and *H. sapiens* microRNAs

The list of miRNAs from *M. oleifera* (733) and immune-related miRNAs from *H. sapiens* (94) were used for feeding MirCompare. Our renovated prediction tool performed a total of 77,362 comparisons, but only 6 passed the filtering phase and were selected as *M. oleifera* microRNAs that exhibit functional homology with their human counterparts (Supplementary Table 2). Notably, previous studies have demonstrated that plant miRNAs have acted in mammalian fashion at least once in the host environment^72^, and this premise simplifies the manner in which target genes are predicted. MirCompare identified a list of 1266 human genes that are predicted to be directly targeted by *M. oleifera* microRNAs (Supplementary Table 3).

**Figure 6:**
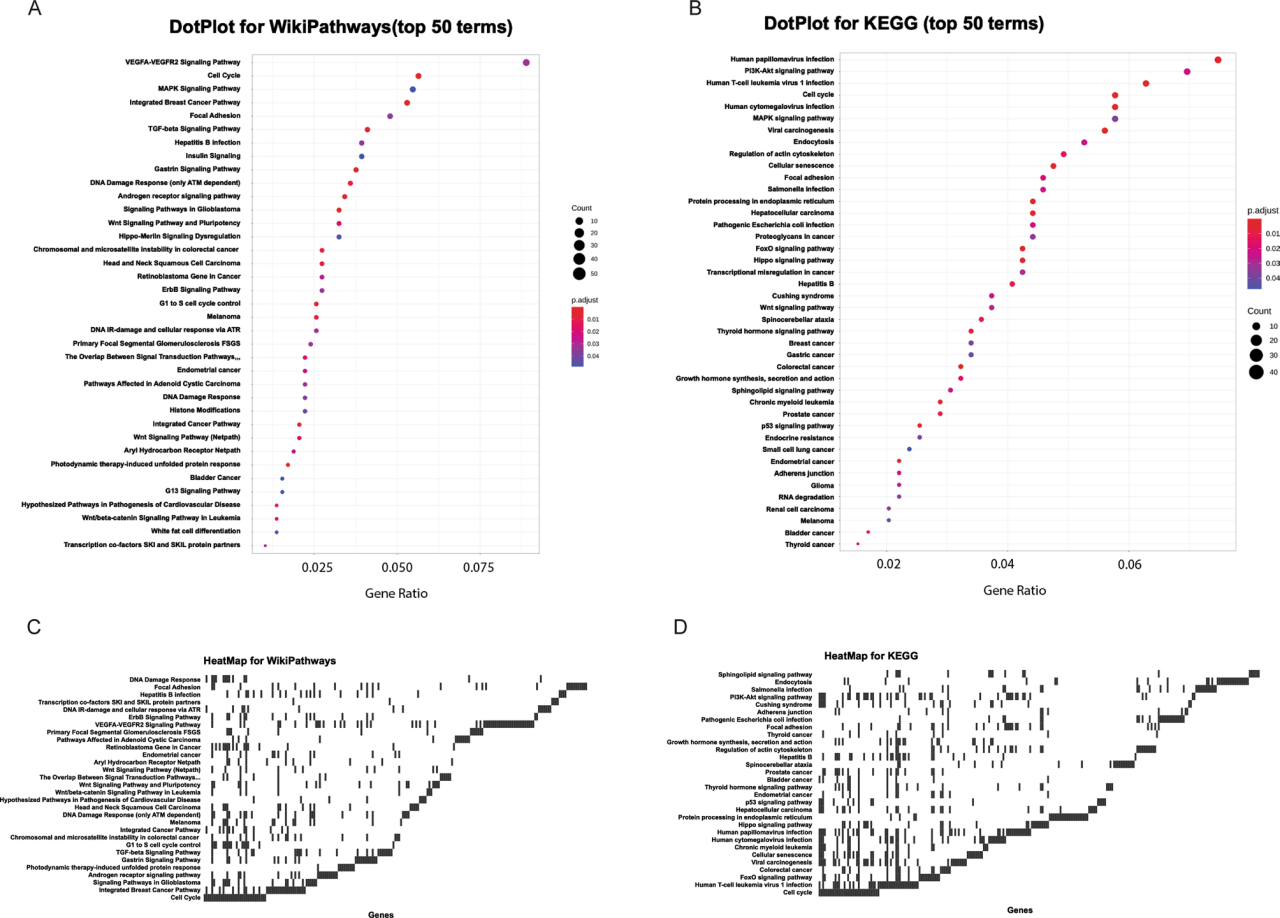
WikiPathway^49^ (**A**,**C**) and KEGG^15^ (**B**,**D**) enrichment analyses for *H. sapiens* genes, putatively targeted by *M. oleifera* microRNAs.

We then applied the enrichment feature to understand which biological processes and functions are overrepresented. As shown in Fig. 6A and B, both WikiPathways and KEGG databases highlight host-virus infection as main terms, with a particular focus on hepatitis B, coronavirus, papillomavirus and cytomegalovirus. Looking at the corresponding heat maps (Fig. 6C,D), integrins (ITGs) clustered together by many of the aforementioned terms (Supplementary Table 4). In accordance with the literature, integrins have been shown to usefully serve as entry receptors for many viruses. RGD (Arg-Gly-Asp) is one of the most common integrin-recognition motifs that interacts with more than 10 different integrins to infect host cells^73^.

## Discussion

MicroRNAs are a class of small (18–24 nucleotides) noncoding RNAs that posttranscriptionally regulate gene expression by interacting with mRNAs. During the last decade, microRNA-mediated processes have emerged to be among the hottest topics in the medical and biological sciences. An impressive number of publications proved the strong association of miRNAs and critical biological events such as inflammation, apoptosis, and carcinogenesis^74–80^. High-throughput technologies have produced an increasing amount of experimental and biomedical data that are difficult and extremely time-consuming for researchers to mine for the correct information flow and to extract new biological insights.

Encouraged by the exponential growth of interest in microRNAs, their epigenetic regulation and all the aforementioned implications, we created MAP, the MicroRNAs Analytics Portal.

The primary focus of MAP is to provide users with a set of bioinformatics analyses on molecular/sequencing data that, otherwise, would be difficult and time-consuming to retrieve manually. At the time of this writing, MAP represents the richest, most complete and integrated database focused on microRNAs. Such a powerful combination of functionalities and analytical modules (general and cancer-specific) makes it possible to address challenging problems quickly and easily.

We tested MAP by selecting microRNA in *M. oleifera* that may be involved in cross-kingdom regulation of immune genes in *H. sapiens.* Starting from a literature-based, exploratory analysis focused on the “microRNAs and immunity” topic, we selected a total of 94 human microRNAs. MirCompare was then used to select the functional homologous counterparts in *M. oleifera*. Among all the pairwise comparisons (77,363), 6 M*. oleifera* miRNAs were selected for their putative capability to regulate mammalian genes. The prediction of human target genes and a series of functional enrichment analyses highlighted the pivotal involvement of integrins (ITG5/6/8/11/V) in host-virus interaction processes, with a particular focus on hepatitis B, cytomegalovirus, papillomavirus and coronavirus.

Moreover, integrins have a central role in regulating the inflammation process by promoting leukocyte adhesion and subsequent maturation processes^81^.

We speculate that the molecular mechanisms underlying the anti-inflammatory properties of *M. oleifera,* the original subject of our case of use, are based on the selected microRNAs in *M. oleifera* that can target ITG genes, reducing their endogenous expression and overall ITG-dependent inflammatory activity.

Obviously, this hypothesis represents a proof-of-concept, and further experimental analyses need to be carried out to validate and better investigate the veracity of these predictions.

We focused on the capability of MAP to perform simple and complex queries without needing a computational background. Biological insights can be retrieved with just a few clicks, and the risk of being overwhelmed by the massive amount of information is minimal.

The best outcome for us would be our resource becoming a keystone for the scientific community that studies microRNAs, actively helping them accelerate their research projects.

## Supplementary Information

Supplementary Table 1.

Supplementary Table 2.

Supplementary Table 3.

Supplementary Table 4.
